# Laparoscopic Heminephrectomy for Duplex Kidney in Children—The Learning Curve

**DOI:** 10.3389/fped.2019.00117

**Published:** 2019-04-02

**Authors:** Marcin Polok, Agata Dzielendziak, Wojciech Apoznanski, Dariusz Patkowski

**Affiliations:** Department of Pediatric Surgery and Urology, Wroclaw Medical University, Wroclaw, Poland

**Keywords:** heminephrectomy, partial nephrectomy, duplex kidney, children, laparoscopy

## Abstract

**Objectives:** Outcomes evolution during the learning curve of laparoscopic transperitoneal heminephrectomy in children with a duplex kidney.

**Materials and Methods:** The data of 33 children, operated on between 2008 and 2017, with complicated duplex kidney, were subjected to a retrospective analysis. All patients were operated on by transperitoneal access using the laparoscopic technique. Patients were divided into two groups (1: subsequent operation 1–16, and 2: operations 17–33) to evaluate the learning curve.

**Results:** There was no need for conversion. Total complications occurred in seven patients in Group 1 and 2 patients of Group 2 (*p* < 0.05). Two patients (6%) (both upper pole heminephrectomies) required reoperation to remove the ureter stump because of recurrent UTIs due to undiagnosed VUR to the stump (1 from each of Groups 1 and 2). Prolonged urine leakage (over 4 days) was observed in four patients (12%; all from group 1); in three patients, conservative treatment was successful, while the placement of a DJ catheter was needed in the fourth. A significant decrease of kidney function (>6%) in scintigraphy was observed in two of the 15 patients after the surgery. The time of surgery decreased from 140 (range 85–185) min in Group 1 to 125 (range 100–150) min in Group 2 (*p* < 0.05).

**Conclusions:** Laparoscopic heminephrectomy is connected to the learning curve. Most complications occurred in the first 16 operations. With increasing experience, the time of operation decreased. In patients with reflux to the upper pole, referred for upper pole heminephrectomy, it is necessary to consider the removal of the ureter to the level of the vesicoureteral junction.

## Introduction

The laparoscopic technique in urology and pediatric surgery has become very popular in recent years. The first laparoscopic nephrectomy in the adult population was performed in 1991 by Clayman et al. (1). In children, both nephrectomy and nephroureterectomy were successfully performed for the first time in 1993 (2). In the same year, Jordan and Winslow successfully carried out the world's first laparoscopic heminephrectomy in a child (3). The advantages and disadvantages of this method are regularly subjected to many debates. Undoubtedly, a longer operation time and higher costs, as well as a more difficult operating technique compared to the classical technique, are given as the disadvantages of laparoscopic heminephrectomy. Among the advantages are shorter hospitalization time, less postoperative pain, a better cosmetic effect, and faster return to full physical activity in the child (4, 5).

Heminephrectomy in a duplex kidney (two pelvicalyceal systems) is a procedure used for the complicated clinical course. The most common indications for surgery in children are recurrent urinary tract infections (UTIs), ureterocelles with a hypo or a functioning moiety and ectopic ureter causing incontinence in girls. Laparoscopic partial/heminephrectomy is technically demanding and requires a learning curve (6–9). It is very important for both the number of complications and the time of surgery. The objective of the study was outcome evolution during the learning curve of laparoscopic transperitoneal heminephrectomy in children with a duplex kidney.

## Materials and Methods

The data of 33 children, operated upon between 2008 and 2017 in the Department of Pediatric Surgery and Urology of the Wroclaw Medical University due to the duplex kidney, were subjected to retrospective analysis. Patients were divided into two groups to evaluate the learning curve (Group 1: subsequent operations 1–16, and Group 2: subsequent operations 17–33). A learning curve was also assessed for operation time.

The indications for surgery were recurrent UTIs (secondary to VUR and/or associated with an ureterocele) connected with loss of kidney moiety function (<10%) and ectopic ureter causing urinary incontinence. In pre-operative diagnostics, all patients underwent abdominal ultrasonography, voiding cystourethrography (VCUG), and kidney scintigraphy. In 14 patients, the diagnosis was additionally supplemented with a computed tomography (CT) urogram. In one child with diagnosed ureterocele 16 months before surgery, cystoscopy with ureterocele incision was performed. All patients were operated on by transperitoneal access using the laparoscopic technique. The patient was placed in an oblique position, with elevation of the operated side. Through the incision at the lower part of the umbilicus, a 5 mm trocar was inserted into the peritoneal cavity. In addition, two 5 mm trocars were inserted. After incision of the parietal peritoneum, both ureters were identified. The next stage after the dissection of the ureter of the resected moiety was unveiling of the kidney pole and the identification of polar vessels. The vessels supplying the affected kidney pole were either clipped or closed with Bi-clamp or Ligasure bipolar forceps and cut. After the demarcation line was made visible on the kidney, the moiety of the kidney was removed by a diathermy Bi-clamp or Ligasure. In an exception, a monopolar hook was used. The ureter was mobilized to the height of the iliac vessels and excised, leaving an open distal stump. It was only in the case of a ureter with confirmed vesicoureteral reflux that it was cut after ligation close to the bladder wall. The resected moiety of the kidney with the ureter was removed through the umbilicus. All children were left with a drain in the kidney area. After removal of the trocars, single sutures were placed on the fascia and the skin, followed by a sterile dressing.

All patients were subjected to control after the surgery together with ultrasound examination of the urinary tract. Further postoperative care depended on the results of the ultrasound examination and the course of further treatment.

Fisher's exact test with Wessa software and the unpaired *t*-test was used for statistical evaluation in the manuscript.

## Results

The patient demographics are provided below in Table 1. There was no need to convert to an open surgery in any case. The outcomes of all procedures are presented in Table 2. Total complications occurred in seven patients of Group 1 and 2 patients of Group 2 (*p* < 0.05). Prolonged urine leak over 4 days was observed in four patients (12%; all in Group 1). In 3 of them, the leak resolved with a catheter in the bladder. In one patient, a double-J (DJ) catheter was placed on the 11th postoperative day using cystoscopy. The leak stopped after 2 days, after 6 weeks the DJ catheter was removed. Two patients required reoperation to remove the ureter stump (one each from Groups 1 and 2) (both upper pole heminephrectomies). Both had recurrent UTIs in the course of the vesicoureteral reflux to the stump that was not visualized on preoperative cystourethrography. The procedure was performed using a laparoscopic technique. These complaints disappeared in both patients. Urinary tract infections after surgery were observed in another two patients. In both, UTI resolved after conservative treatment (antibiotics). In the ultrasound image, no significant pathology was found in 32 patients, and 1 cyst of the lower pole of the kidney persisted. In a postoperative renoscintigraphy performed in 15 patients (45.45%), two patients (one each from Groups 1 and 2) showed a decrease in the function of the residual moiety (from 51 to 26% and from 43 to 27%) compared to the baseline study. Total complications occurred in seven of 28 (25%) patients of upper pole heminephrectomies and in two of five (40%) of lower pole heminephrectomies (Table 2). Outcomes depending on the surgeon's experience are presented in Table 3. The decreasing operation time in subsequent procedures as a “learning curve” is given in Table 4.

**Table 1:** Patient demographics before surgery.

**Median age (years)**	**3,5 (range 2 months−16 years)**	
Sex (female, male)	Female-26	Male-7
Operated kidney (right/left)	Left-21	Right-12
Recurring urinary tract infections (yes/no)	Yes-32	No-1
Incontinence (yes/no)	Yes-3	No-30
Vesicoureteral reflux (yes/no)	Yes-8	No-25
Ureterocele (yes/no)	Yes-1	No-32
Kidney moiety that needs to be removed (Upper/lower)	Upper-28	Lower-5

**Table 2:** Outcomes of all procedures.

**Operation time (min.)**	**85–185 (median 137)**
Drain after operation (days)	1–12 (median 2)
Painkillers after operation (days)	2–12 (median 2)
Hospitalization (days)	2–14 (median 4)
Reoperation to remove the ureter stump	2 (6%) (all UPHN)
Prolonged urine leak for more than 4 days	4 (12%) (2-UPHN, 2-LPHN)
Cyst in ultrasound	1 (3%) (UPHN)
Decrease of the kidney function (DRF) in scintigraphy (was done in 15 of 33 patients after the surgery)	2 of 15 (13.3%) (all UPHN)
Median follow up (years)	3,5 (range 1–9.5)

*UPHN - upper pole heminephrectomy; LPHN - lower pole heminephrectomy*.

**Table 3:** Comparison of outcomes depending on the surgeon's experience (learning curve).

	**Group 1 (Patients 1–16)**	**Group 2 (Patients 17–33)**	**Statistical significance (*p*-value)**
Total complications (a–d)	7	2	0.0391
a. Prolonged urine leak over 4 days	4	0	0.0445
b. Reflux to the ureter stump	1	1	1
c. Kidney cyst in ultrasound	1	0	0.4848
d. Decrease in the function (DRF) of the residual moiety over 6%	1	1	1
Median operation time (minutes)	140 (range 85–185)	125 (range 100–150)	0.0031
Median age (months)	63 (range 2–200)	22 (range 4–75)	0.0256

**Table 4:**
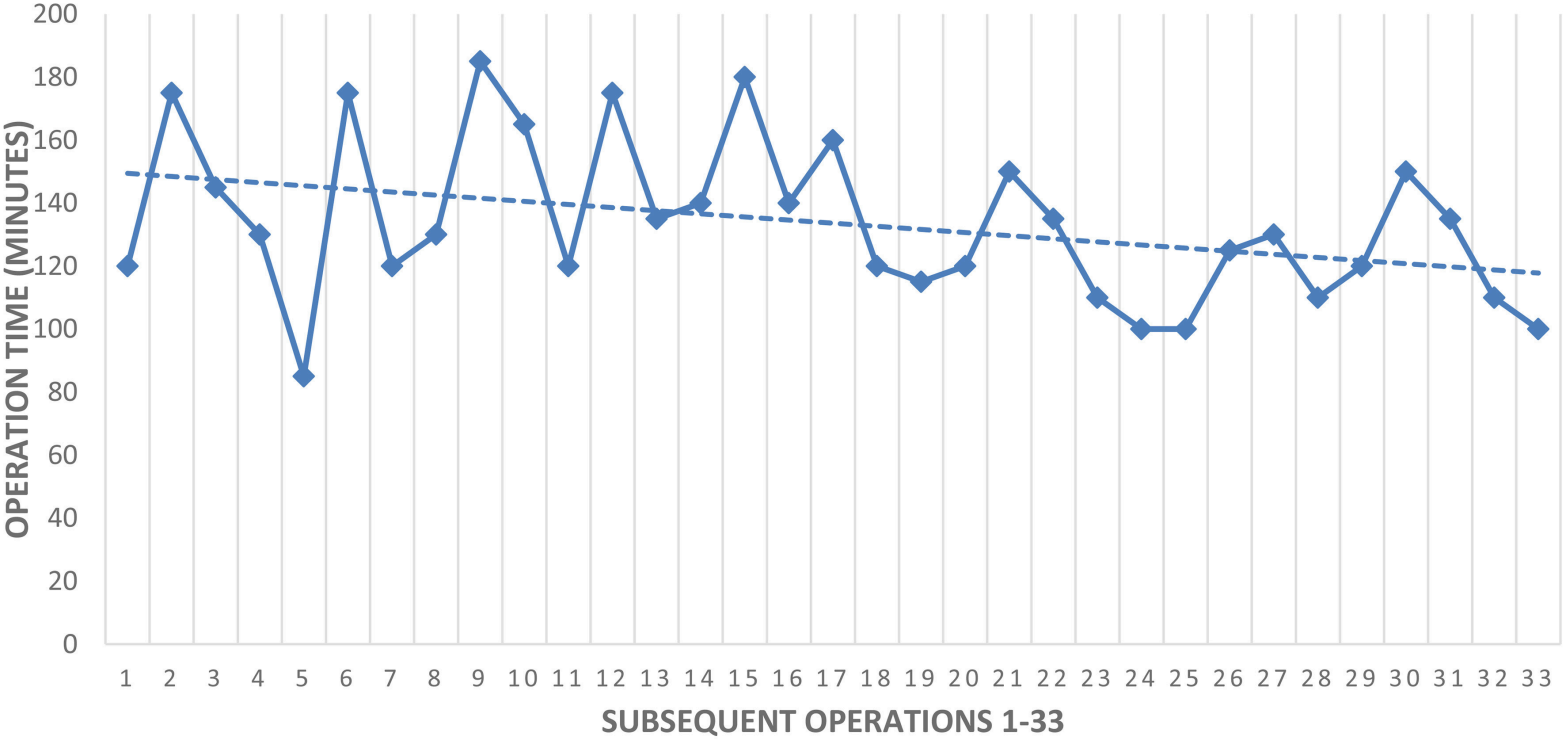
Operation time in subsequent procedures as a learning curve.

## Discussion

Experience in minimal invasive surgery (MIS) has always been considered one of the most important factors in accomplishing continuous, successful outcomes. Laparoscopic partial/heminephrectomy is technically difficult and has a steep learning curve (6–9). It is highly important for conversion rates, the number of complications and time of surgery. Leclair et al. reported conversion to open surgery, using retroperitoneal access, in ten (21%) of 48 patients (9). The authors concluded that the incidence was clearly related to experience with a rate of 1 in 20 (5%) during the last 20 procedures, compared to 8 in 20 (40%) during the first 20. They reported having difficulties during parenchymal section with bipolar diathermy in the first series, while these problems resolved with the acquisition of the Harmonic scalpel.

Additionally, the retroperitoneal access seems to be burdened with a higher conversion rate and a higher rate of complications. In our study, we used only transperitoneal access. In no case was it necessary to convert the operation to open surgery. In our opinion, transperitoneal access gives better visualization and a greater chance of avoiding damage to the vessels. Other authors using this access also report not performing any conversions to open surgery (10–12).

In our material, total complications occurred in seven patients in the first 16 procedures and only in 2 in last 17. Four children had prolonged urine leakage after surgery lasting for over 4 days. In these patients, prolonged drainage was necessary with an extended hospital stay. Other authors also encountered this complication (7, 10). It appears that prolonged urine leakage occurs in patients who had a duplex kidney pelvicalyceal system of the residual moiety opened or a ureteral stump that was not closed. In our sample, the ureteral stump was left open in 25 patients (75%). However, a Foley catheter in the bladder was used in all patients after the operation. Therefore, leakage from the bladder seems unlikely; in our opinion, the leak was from the remaining renal moiety. All cases of prolonged urine leakage (*n* = 4) occurred in the first series of 16 patients. Along with gaining experience (patients 17–33), the above complications did not occur. These results can be clearly linked to the surgeon's learning curve.

Two of our patients (6%) required reoperation due to recurrent UTI in the course of reflux to the residual ureter stump. In both patients upper pole heminephrectomy was done. Other authors also met with similar complications (13, 14). Sydorak and Shaul (14) reported this complication in 1/9 (11.1%) of patients undergoing transperitoneal heminephrectomy. They emphasized that this complication could have been avoided if the entire ureter had been removed and tied at the entrance to the bladder. In our patients, we removed the entire ureter in each case when vesicoureteral reflux (VUR) was confirmed in the cystography. In both cases of VUR to the stump, it was not originally present. Considering such experience, it may be possible for some patients after surgery to change the parameters of their bladder function and the occurrence of VUR. In such cases, the removal of the entire ureter should be taken into consideration, regardless of whether we deal with the VUR. Barroso et al. performed 16 total and 9 partial, open nephrectomies in 87% without ureteral stump removal. They concluded that for primary vesicoureteral reflux there is no need of ureteral stump removal. However, when there is an underlying pathology such as ureterocele or ectopic ureter, it might be the case (15).

In two of the fifteen patients who had a diuretic renography after surgery, there was a decrease in function of the remaining moiety. In both cases, because of a long wait for the surgery in hospital, the preoperative radionuclide scan should not be considered reliable. This study was performed exactly 6 and 9 months before the surgery, so the real function of the duplex kidney at the time of operation was probably much lower. The same complication was reported by Wallis et al. (16) in two of fifteen patients who had functional scans and by El Ghoneimi et al. (8) in one patient. Leclair et al. (9) described the loss of function over 5% in 7 (24.13%) of nuclear scans after surgery. The authors concluded that loss of function could occur as a result of inadvertent stretching and subsequent stenosis of the vascular pedicle to the remaining moiety.

The time required for surgery also depends on the surgeon's experience. In our material, the median operation time was 137 min. With increasing experience, the time of operation was significantly decreased, with surgery lasting for 140 min in Group 1 and only 125 min in Group 2. This time is clearly shorter than is commonly reported in the literature, and may be associated with the extensive experience in minimally invasive surgery of the operating team. In literature reports, the time of heminephrectomy surgery from transperitoneal access ranges from 167 to 190 min (7, 10, 11, 17, 18). Obtaining experience is further proved in a report by Lee et al. The authors performed the first heminephrectomy using a da Vinci robot in nine pediatric patients (19). The average time of surgery was much longer than in the mentioned laparoscopic operations, reaching 275 min on average, and was even 417 min in one case. Moreover, 33.3% of patients had complications (urinoma, umbilical hernia).

Laparoscopic heminephrectomy is connected to the learning curve. Most complications occurred in the first 16 operations. With increasing experience, the time of operation decreased. In patients with reflux to the upper pole, referred for upper pole heminephrectomy, it is necessary to consider the removal of the ureter to the level of the vesicoureteral junction.

## Data Availability

All datasets generated for this study are included in the manuscript and/or the supplementary files.

## Ethics Statement

Written informed consent was obtained from all participants. This study was reviewed and approved by the Medical University of Wrocław Ethics Committee (Ethics board approval number: KB - 339/2018).

## Author Contributions

MP and DP contributed conception and design of the study. AD and MP organized the database. MP and AD performed the statistical analysis. MP wrote the first draft of the manuscript. WA and DP wrote sections of the manuscript. All authors contributed to manuscript revision, read and approved the submitted version.

### Conflict of Interest Statement

The authors declare that the research was conducted in the absence of any commercial or financial relationships that could be construed as a potential conflict of interest.
